# A Systematic Review on the Role of Repeat Transurethral Resection after Initial en Bloc Resection for Non-Muscle Invasive Bladder Cancer

**DOI:** 10.3390/jcm11175049

**Published:** 2022-08-28

**Authors:** Henglong Hu, Mengqi Zhou, Binrui Yang, Shiwei Zhou, Zheng Liu, Jiaqiao Zhang

**Affiliations:** 1 Department of Urology, Institute of Urology, Tongji Hospital, Tongji Medical College, Huazhong University of Science and Technology, No. 1095 Jiefang Avenue, Wuhan 430030, China; 2 College of Life Science and Technology, Huazhong University of Science and Technology, 1037 Luoyu Road, Wuhan 430074, China

**Keywords:** bladder cancer, repeat transurethral resection, re-resection, restage, en bloc resection, systematic review

## Abstract

International guidelines recommend repeat transurethral resection of bladder tumors (reTURB) for selected patients with high-risk non-muscle invasive bladder cancer to remove possible residual tumors, restage tumors and improve the therapeutic outcome. However, most evidence supporting the benefits of reTURB is from conventional TURB. The role of reTURB in patients receiving initial En bloc resection of bladder tumor (ERBT) is still unknown. PubMed, Embase, Web of Science, The Cochrane Library, and China National Knowledge Infrastructure (CNKI) were systematically searched. Finally, this systematic review and meta-analysis included twelve articles, including 539 patients. The rates of residual tumor and tumor upstaging detected by reTURB after ERBT were 5.9% (95%CI, 2.0%–11.1%) and 0.0% (95%CI, 0.0%–0.5%), respectively. Recurrence-free survival, tumor recurrence and progression were comparable between patients with and without reTURB after initial ERBT. The pooled hazard ratios of 1-year, 2-year, 3-year and 5-year recurrence-free survival were 0.74 (95%CI, 0.36–1.51; *p* = 0.40), 0.76 (95%CI, 0.45–1.26; *p* = 0.28), 0.83 (95%CI, 0.53–1.32; *p* = 0.43) and 0.83 (95%CI, 0.56–1.23; *p* = 0.36), respectively. The pooled relative risks of recurrence and progression were 0.87 (95%CI, 0.64–1.20; *p* = 0.40) and 1.11 (95%CI, 0.54–2.32; *p* = 0.77), respectively. Current evidence demonstrates that reTURB after ERBT for bladder cancer can detect relatively low rates of residual tumor and tumor upstaging and appears not to improve either recurrence or progression.

## 1. Introduction

Bladder cancer is among the world’s top ten most common cancer types, with approximately 550,000 new cases annually [1,2]. Non-muscle-invasive bladder cancer (NMIBC), which includes Ta, T1, and carcinoma in situ, represents approximately 75% of all bladder cancers at initial diagnosis [3]. Transurethral resection of the bladder (TURB) is the standard procedure for bladder cancer diagnosis and represents, at the same time, the most important therapeutic moment for patients with NMIBC [3]. Although conventional TURB (cTURB) is widely used and has piled tremendous expertise over decades, multiple drawbacks are still associated with it. Such issues are, for example, tumor cell scattering through fragmentation, the risk of tumor cell seeding and reimplantation, a rather high rate of missing detrusor muscle (DM) and downstaging, thermal damage of sensitive areas within the specimens, and incomplete resections [4].

To overcome these drawbacks of cTURB, En bloc resection of bladder tumor (ERBT) and second or repeat TURB (reTURB) have been introduced to clinical practice [4]. ERBT applies a novel technique to cTURB, resecting the entire tumor, the surrounding mucosa, the underlying stroma, and superficial muscularis propria in a single specimen [5]. Recently, there has been increasing evidence to support the clinical benefit of ERBT. Compared to cTURB, ERBT has a higher DM presence rate, seems safer, and yields superior histopathologic information and performance [6,7]. ERBT is most feasible for patients with bladder tumor size of ≤3 cm. For bladder tumor size of >3 cm, the specimen may not be retrieved in one piece. However, the resection procedure itself is still technically possible, and the potential benefits can still be preserved [8].

An early reTURB is recommended to be performed for selected patients by all the most followed international guidelines in the urological community (Table 1) [3,9,10,11,12,13,14,15]. Compared with initial TURB, reTURB can remove the residual tumors, detect understaging BC, improve the responsive rate of intravesical Bacillus Calmette-Guerin (BCG) instillation, and instruct further treatments [16,17,18,19]. A recent study corroborated the important role of routine reTURB, followed by an adequate maintenance course of BCG in organ-sparing NMIBC patients [20]. Interestingly, reTURB was found to be associated with longer recurrence-free survival (RFS) in patients receiving TICE strain maintenance therapy than those using Connaught and RIVM [20,21]. However, it should be underlined that reTURB, which must be done on a patient who may still be suffering from the consequences of the last surgery, is an invasive and morbid technique that significantly lowers the patient quality of life. In addition, it increases the economic burden of bladder cancer care [22]. Moreover, there is no complete agreement in international guidelines as to which patients should be recommended for reTURB surgery, and these recommendations do not consider the impact of the surgical approach (Table 1) [3,9,10,11,12,13,14]. That is why we must further clarify which patients benefit most from reTURB. Currently, most evidence supporting the benefits of reTURB is based on patients receiving previous cTURB [17]. Whether reTURB can improve the outcomes of patients receiving initial ERBT and whether reTURB can be safely avoided by ERBT patients is still unclear. Therefore, we set out to perform this systematic review and meta-analysis.

## 2. Materials and Methods

### 2.1. Literature Search and Study Selection

The Preferred Reporting Items for Systematic Reviews and Meta-Analyses (PRISMA) statement was followed by our study [23]. The protocol of this study has been registered in Open Science Framework Registry (Registration DOI:10.17605/OSF.IO/9FWVM). PubMed, Embase, Web of Science, The Cochrane Library, and China National Knowledge Infrastructure (CNKI) were systematically searched to identify relevant studies. The search was first performed on 30 April 2022 and updated on 12 July 2022. The initial search process was designed to find all relevant published original articles without limitation by year or language. Detailed search terms were: (repeat* [Title/Abstract] OR second [Title/Abstract] OR re-resect* [Title/Abstract] OR re-transurethral [Title/Abstract] OR restag* [Title/Abstract] OR reTUR* [Title/Abstract] OR re-look [Title/Abstract]) AND (“en bloc” [Title/Abstract] OR “en-bloc” [Title/Abstract] OR “enbloc” [Title/Abstract] OR “ERBT” [Title/Abstract] OR enucleate* [Title/Abstract] OR “one piece” [Title/Abstract]) AND (“bladder cancer” [Title/Abstract] OR “bladder tumor” [Title/Abstract] OR “bladder carcinoma” [Title/Abstract] OR “Urothelial carcinoma” [Title/Abstract]). Initial screening was performed independently by two investigators (Dr. Henglong Hu and Dr. Jiaqiao Zhang) based on the titles and abstracts to identify eligible reports. Potentially relevant reports were subjected to a full-text review. Disagreements were resolved by consensus with the co-investigators.

### 2.2. Inclusion and Exclusion Criteria

We focused on the reTURB outcomes after ERBT, such as residual tumors, upstage, short-term or long-term recurrence and progression. All kinds of study designs, such as randomized control trials (RCTs), cohort studies and single-arm studies, would be included as long as they reported at least one of the interesting outcomes. However, studies lacking original or necessary data, reviews, letters, conference abstracts, editorial materials, replies from authors, case reports, and patent records were excluded. Studies were excluded if the number of participants was less than five, as they were deemed methodologically inappropriate. In cases of duplicate publications or duplicate data, the study of higher quality or the most recent publication was selected. Disagreements were resolved through discussions.

### 2.3. Data Extraction and Study Quality Assessment

Two investigators extracted the following data from each eligible study independently: first author’s name, publication journal and year, countries, study design, study period, sample size, participants’ characteristics (age, gender), tumor characteristics, en bloc method, reTURB criteria, intravesical therapy, perioperative complications, recurrence and progression status, recurrence-free survival (RFS), progression-free survival (PFS), overall survival (OS) and cancer-specific survival (CSS). Disagreements between the two authors will be resolved by rechecking the articles and discussion. The methodological quality of cohort studies was evaluated using the Newcastle-Ottawa Scale (NOS) for non-randomized controlled trials [24]. The NOS comprises three domains, including participant selection (points range: 0–4), comparability between groups (points range: 0–2), and clinical outcomes (points range: 0–3). NOS scores ≥ 6 indicate high methodological quality. For single-arm studies and studies in which we only retrieved one arm data, a five-criterion quality appraisal checklist proposed by the European Association of Urology Guidelines Office was used [25]. The five aspects included: 1. Was there an a priori protocol? 2. Was the total population included or were study participants selected consecutively? 3. Was outcome data complete for all participants, and was any missing data adequately explained/unlikely to be related to the outcome? 4. Were all prespecified outcomes of interest and expected outcomes reported? 5. Were primary benefit and harm outcomes appropriately measured? If the answer to all five questions is “yes,” the study is at a “low” risk of bias. If the answer to any question is “no”, the study is at a “high” risk of bias [25]. Possible publication bias was assessed using funnel plots, Egger test, and Begg’s test.

### 2.4. Data Processing and Statistical Analysis

Dichotomous variables were reported by counts and percentages, while continuous variables were reported as mean± standard difference or median ± interquartile range (IQR: 25th and 75th) or range. The impact of reTURB on survival and disease control was measured by the effect size of the hazard ratio (HR), RFS, PFS, OS, and CSS. They were extracted directly from each study if reported by the authors. Otherwise, these data were estimated indirectly using the method described by Tierney et al. [26]. Each study’s Kaplan–Meier plots were downloaded and digitized using the GetData Graph Digitizer (version 2.26; http://getdata-graph-digitizer.com/index.php; accessed on 1 July 2022), and survival probabilities at different follow-up times were extracted. Then, the number of subjects at risk, adjusted for censoring at different follow-up times, was calculated to reconstruct the HR estimate.

The statistical analysis and meta-analysis were performed using STATA version 17.0 software (StataCorp, College Station, TX, USA). A *p*-value less than 0.05 was considered statistically significant. Heterogeneity among studies was evaluated by the chi-square test, *I*^2^ statistics, and Galbraith plots. Moreover, the pooled estimates were calculated with the fixed-effect model if no significant heterogeneity was detected; otherwise, the random-effect model was used. The z-test determined the pooled effects. As mentioned above, funnel plots were generated to assess any bias, and both the Egger and Begg’s tests were done to examine any statistical significance of publication bias. If there is a significant publication bias or pooled studies of less than five, a sensitivity analysis was performed using the trim and fill method to test the robustness of the results.

## 3. Results

### 3.1. Literature Search and Study Selection

**Figure 1:**
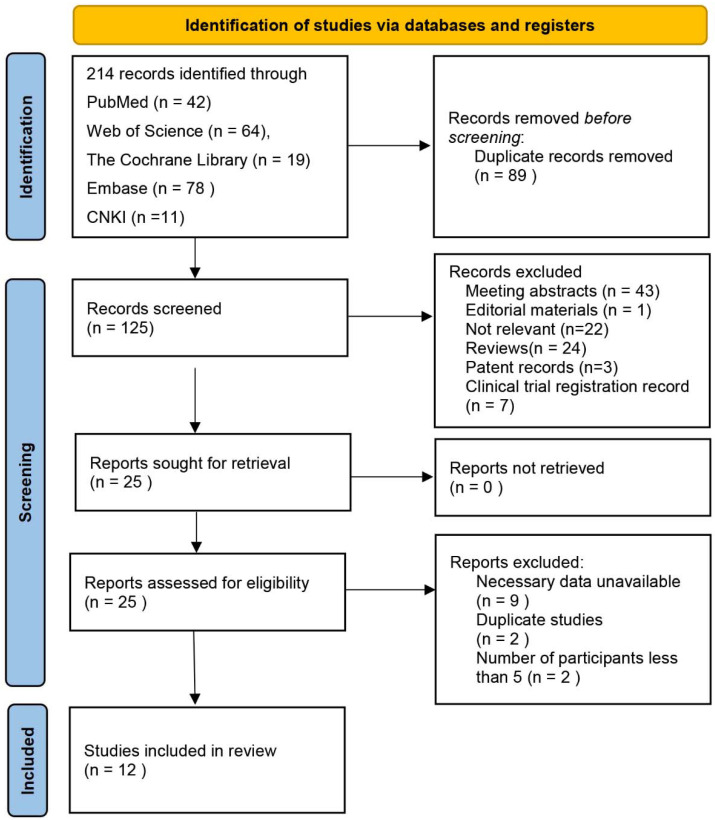
Flowchart of the studies selection process. CNKI: China national knowledge infrastructure.

Figure 1 shows the process of literature search and study selection. Electronic searches of five databases revealed 214 records. After screening titles and abstracts, we found 25 articles relevant to the study aim, and therefore we retrieved the full-text articles. After full-text analysis, another 13 studies were excluded for the following reasons: nine lacked necessary data, two reported duplicated data, and only two studies only reported one patient. Finally, 12 studies fulfilled our eligibility criteria and were enrolled in this review [27,28,29,30,31,32,33,34,35,36,37,38].

### 3.2. Systematic Reviews of Included Studies

Table 2 summarizes the characteristics of the 12 eligible studies published from 2011 to 2022. Five of the studies were conducted in China [31,32,34,36,37], three in Italy [28,29,30] and one each in Egypt [33], Germany [27], Japan [38], and Poland [35]. All these studies were conducted in the last 12 years. Most patients included were high-risk patients with high-grade and/or tumors. Some studies had limited the tumor size to less than 3 cm or 4 cm. Some early studies only included single tumor patients to facilitate the en bloc resection, and recent studies had no limits or limited the neoplasm number to no more than 3 or 4. The re-resection time was relatively consistent, most of them were performed within 6 weeks after the initial resection. There are three cohort studies that directly compared patients who received reTURB after ERBT with those who only underwent ERBT [32,36,38]. All these studies were published in the last two years which indicates that this topic has recently gained the attention of researchers and is gradually becoming popular. There are six single arm studies that reported the outcome of reTURB after ERBT. Although the objectives of two cohort studies and one RCT were to compare ERBT with cTURB, the data of the ERBT arm of the three studies were also retrieved and analyzed.

### 3.3. Residual Tumors and Upstage at reTURB after ERBT

**Figure 2:**
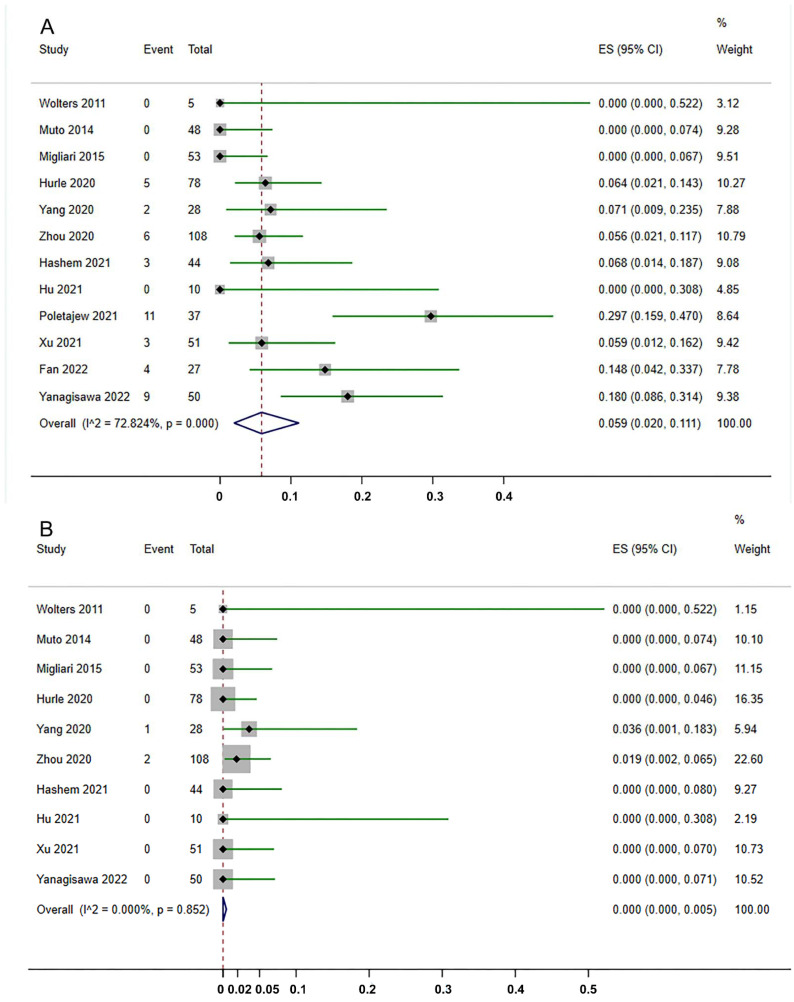
Forest plots of the rates of residual tumor (**A**) and tumor upstaging (**B**) detected by reTURB after initial ERBT [27,28,29,30,31,32,33,34,35,36,37,38]. ES: effect size. The dash lines represent the pooled effect size.

All 12 studies reported the status of residual tumor after ERBT. The residual tumor rate varied from 0% to 29.3%. As shown in Figure 2A, pooling the data from 539 patients demonstrated that the residual tumor rate detected by reTURB after ERBT was 5.9% (95%CI, 2.0%–11.1%). Only one study reported the residual tumor location [38]. Among 50 patients, six had residual tumors at the original site, while two were at the non-original site. Ten studies revealed the upstaging rate at reTURB after ERBT ranged from 0% to 3.57%. Surprisingly, as shown in Figure 2B, the meta-analysis demonstrated that the upstaging rate at reTURB is 0.0% (95%CI, 0.0%–0.5%).

### 3.4. Recurrence and Progression

**Figure 3:**
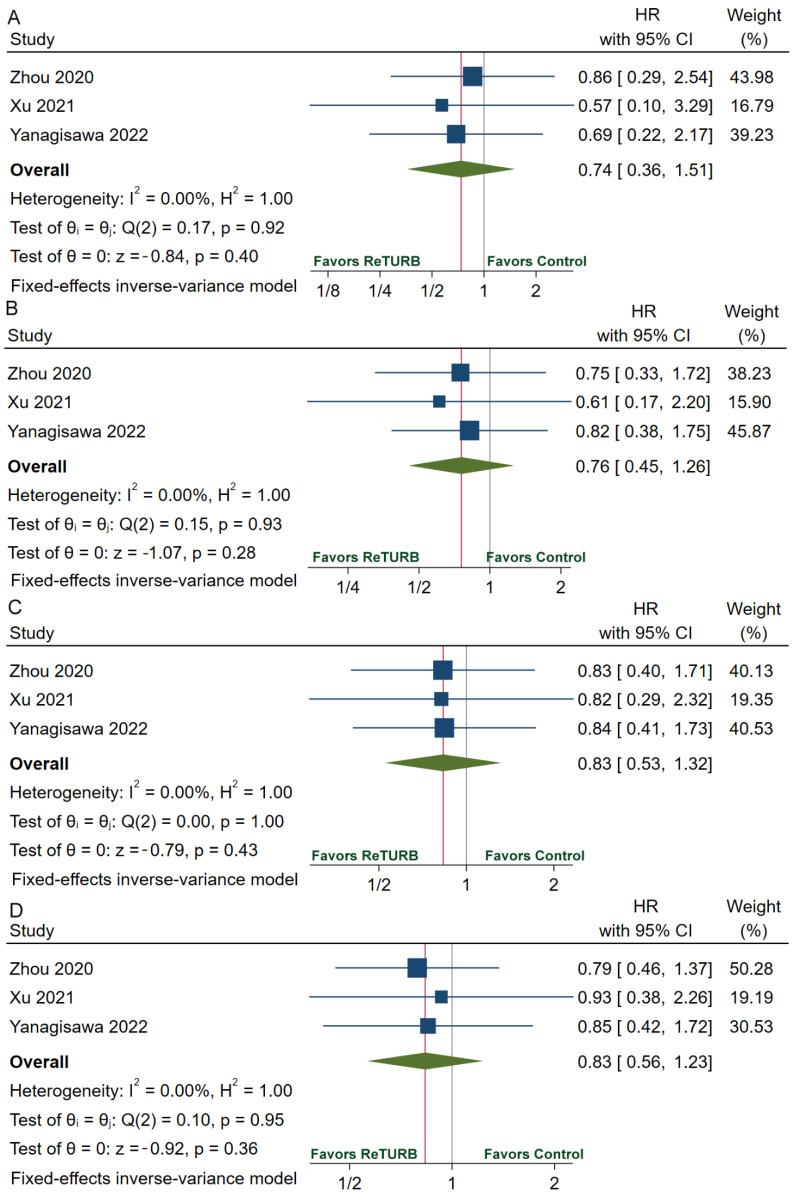
Forests plots of comparisons of 1-year RFS (**A**), 2-year RFS (**B**), 3-year RFS (**C**), and 5-year RFS (**D**) between the reTURB group and control group [32,36,38]. The gray lines represent the reference lines and the red lines show the pooled effect sizes. RFS: recurrence-free survival; HR: hazard ratio; reTURB: repeat transurethral resection of bladder tumors; CI: confidence interval.

**Figure 4:**
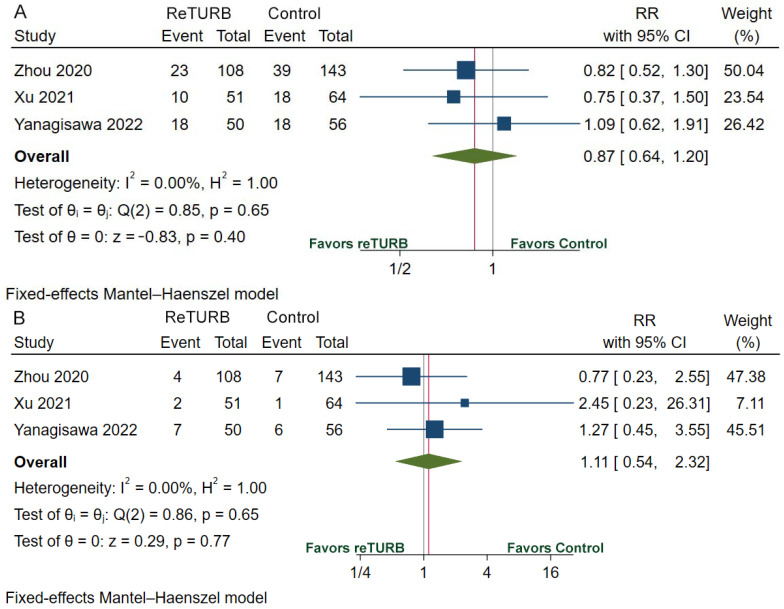
Forests plots of comparisons of recurrence (**A**) and progression (**B**) risk between the reTURB group and control group [32,36,38]. The gray lines represent the reference lines and the red lines show the pooled effect sizes. CI: confidence interval. RR: relative risk; reTURB: repeat transurethral resection of bladder tumors.

Table 2 provides the recurrence, progression, RFS, and PFS data. The recurrence rate ranges from 14.1% to 36.0% in the reTURB group and 27.2% to 32.1% in the patients who did not receive reTURB. RFS was comparable between patients with and without reTURB after initial ERBT. The pooled HRs of 1-year, 2-year, 3-year and 5-year RFS were 0.74 (95%CI, 0.36–1.51; *p* = 0.40), 0.76 (95%CI, 0.45–1.26; *p* = 0.28), 0.83 (95%CI, 0.53–1.32; *p* = 0.43) and 0.83 (95%CI, 0.56–1.23; *p* = 0.36), respectively (Figure 3). The pooled relative risk (RR) of recurrence was 0.87 (95%CI, 0.64–1.20; *p* = 0.40) (Figure 4A). The progression rate ranged from 0.0% to 14.0% in the reTURB group and 1.5% to 10.7% in the control group. Meta-analysis reveals that RR of progression was 1.11 (95%CI, 0.54–2.32; *p* = 0.77) (Figure 4B). No study reported the outcomes of OS and CSS.

### 3.5. Risk of Bias Assessment, Heterogeneity, and Sensitivity Analysis

**Figure 5:**
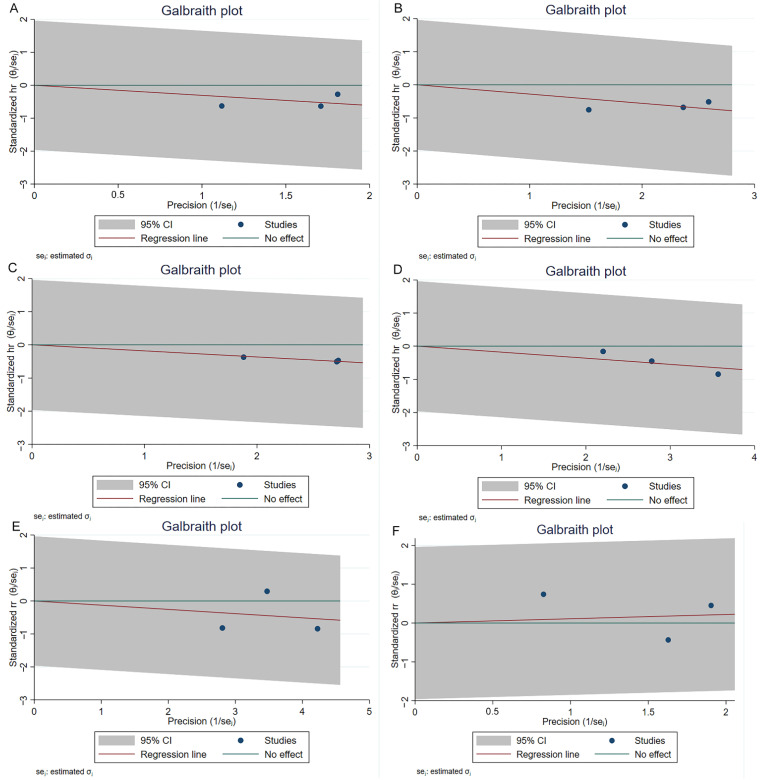
Galbraith plots of comparisons 1-year RFS (**A**), 2-year RFS (**B**), 3-year RFS (**C**), 5-year RFS (**D**), recurrence (**E**) and progression (**F**) between reTURB group and control group. CI: confidence interval.

**Figure 6:**
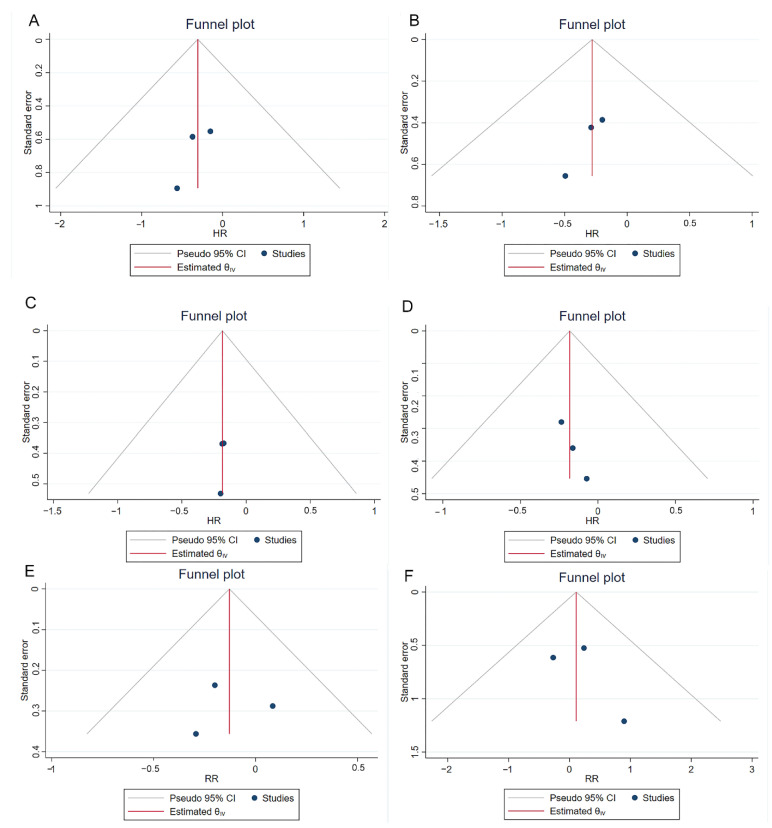
Funnel plots of comparisons 1-year RFS (**A**), 2-year RFS (**B**), 3-year RFS (**C**), 5-year RFS (**D**), recurrence (**E**) and progression (**F**) between reTURB group and control group. CI: confidence interval.

The NOS scores of three cohort studies have been shown in Table S1, and the quality of these three studies was considered high. All the other studies except for the RCT article have been assessed by the five-criterion quality appraisal checklist and consider to be at high risk of bias (Table S2). Heterogeneity among comparative studies was evaluated by the chi-square test, *I^2^* statistics, and Galbraith plots (Figure 5). No significant heterogeneity was detected. Although no significant publication bias was found in the funnel plot (Figure 6), Egger test, and Begg’s test (Table S3). We also performed a sensitivity analysis. The sensitivity analysis using the trim and fill method generated similar results, which indicated these pooling results were stable and reliable (Table S3). Figure S1 shows the funnel plots of sensitivity analysis.

## 4. Discussion

The cTURB represents the most important endoscopic treatment of bladder tumors. However, cTURB’s oncological outcomes have been doubted, given the high residual disease and recurrence rates [4]. For instance, residual tumor at re-resection has been shown in 17–67% of Ta and 20–71% of T1 diseases [39]. Apart from the high incidence of residual and recurrent tumors, cTURB is limited by the risk of understaging due to the absence of DM layer in the specimen, as the presence of DM is a surrogate marker of resection quality which strongly determines prognosis [4,40,41]. An early reTURB is recommended for selected patients to remove any residual disease, restage the tumor and improve the therapeutic outcome. However, most of the previous evidence is based on initial cTURB. Recently, ERBT has emerged as an alternative to cTURB [42]. In contrast to ‘piecemeal’ resection by cTURB, ERBT incorporates a more delicate en bloc sculpting and tumor excision [43]. ERBT appears safe, feasible, and effective with demonstrably higher rates of DM in the pathologic specimen and provides better staging [6]. Given the excellent quality of the initial resection provided by ERBT and evidence supporting the completeness of tumor resection and reduced residual disease, ERBT might result in less need for reTURB. Therefore, we performed this systematic review to analyze the impact of reTURB on patients who underwent initial ERBT. 

A comprehensive review and meta-analysis demonstrate that the residual tumor rate detected by reTURB after ERBT is 5.9% (95%CI, 2.0–11.1%), and the upstaging rate is 0.0% (95%CI, 0.0–0.5%). Residual tumor at reTURB after cTURB has been described in up to 75% of Ta and T1 patients [39]. Even more profound is the rate of upstaging from Ta to T1 or T1 to T2 at reTURB, which has been observed in up to 28% of initial T1 and 9.5% of initial TaHG tumors, respectively [39]. A recent meta-analysis finds that the residual and upstaging rates of T1 BC in reTURB were around 50% and 10%, respectively [44]. All of these are much higher than that of patients who underwent ERBT. If we still do not take the surgical method into account and choose the real “high risk” patients, more patients will take an “unnecessary” reTURB at the risk of perioperative complications and raising the already high cost [45].

In addition, our study shows that RFS was comparable between patients with and without reTURB after initial ERBT. The pooled RRs of recurrence and progression were 0.87 (95%CI, 0.64–1.20; *p* = 0.40) and 1.11 (95%CI, 0.54–2.32; *p* = 0.77), respectively. The two groups have comparable 1-year, 2-year, 3-year, and 5-year RFS. ReTURB seems not to benefit patients who underwent initial ERBT in reducing recurrence and progression. However, a recent meta-analysis demonstrated that short-term RFS (1-year and 3-year) of the reTURB group was better compared with the TURB group. The pooled RR were 1.10 (95%CI: 1.01 × 10^1.19^) and 1.15 (95%CI: 1.03–1.28), respectively [44]. While reTURB did not improve long-term RFS (5-year, 10-year, 15-year) in T1 patients. The pooled RR were 1.12 (95%CI: 0.97–1.30), 1.11 (95%CI: 0.82–1.50) and 1.37 (95%CI: 0.50–3.74), respectively [44]. Nearly all of the included patients had undergone initial cTURB and all the patients with T1 tumors. We cannot do a T1 tumor subgroup analysis as lacking relevant data. But one study included in our review find that the 2-year RFS and 3-year PFS were comparable between patients with T1 tumors who underwent reTURB and those who did not (55.1% vs. 59.9%, *p* = 0.6, 80.6% vs. 82.6%, *p* = 0.6, respectively) [38]. No patient was upstaged to pT2 on reTURB. A reTURB after ERBT for pT1 bladder cancer appears not to improve either recurrence or progression [38].

This study has several limitations. First, the number of included studies and recruited patients in some studies was relatively small. We performed the sensitivity analysis to improve this aspect partially, and the stable results from the sensitivity analysis strengthen our conclusion. There is still no RCT directly investigating the impact of reTURB on the patients receiving ERBT. More studies are urgently needed to clarify this clinical problem further. Second, the baseline characteristics of patients in different studies are not the same, which may influence the prognosis. For example, patients in different studies have different tumor characteristics and follow-up periods. But few studies provided detailed outcomes for subgroup patients, such as patients with Ta or T1 tumors. We were not able to conduct more subgroup analyses to adjust the effect. Although all of these may increase the heterogeneity and confound the results, we find no significant heterogeneity in the statistical test. Third, single-arm studies have an inherent risk of bias. We used the random model to minimize the effect. Because of these limitations, the results of this study should be interpreted with caution.

## 5. Conclusions

Current evidence demonstrates that reTURB after ERBT for bladder cancer can detect relatively low rates of residual tumor and tumor upstaging and appears not to improve either recurrence or progression. Although the results should be interpreted with caution, our study would assist clinical decisions making when patients who had undergone initial ERBT are informed about the exact effect of reTURB. Further studies are still needed to confirm and clarify the role of reTURB after ERBT.

## Figures and Tables

**Table 1:** ReTURB recommendations across international guideline panels.

Guidelines Body	Version	Recommendation on Suitable reTURB Candidates	Recommendation Strength	ReTURB Period after the Initial Resection
European Association of Urology	2022	1. Incomplete initial TURB, or in case of doubt about the completeness of a TURB; 2. If there is no detrusor muscle in the specimen after initial resection, except for Ta LG/G1 tumors and primary CIS; 3. T1 tumors.	Strong	2–6 weeks
National Comprehensive Cancer Network (NCCN)	Version 2.2022	1. Visually incomplete resection or **high-volume tumor** 2. **TaHG**, **particularly if large**, and/or no muscle in the specimen 3. T1 tumors	2A *	2–6 weeks
European Society for Medical Oncology (ESMO)	2021	1. The initial TURB was incomplete. 2. If no detrusor muscle exists in the specimen on the initial resection, except for Ta LG and CIS. 3. In all pT1 tumors and **all HG tumors**, **except for patients with primary CIS**	Strong	4–6 weeks
Canadian Urological Association	2021	1. Incomplete initial TURB 2. **TaHG tumors (e.g., large and/or multiple tumors)** 3. T1 tumors	1. Strong 2. Weak 3. Strong	within 6 weeks
American Urological Association & Society of Urological Oncology	2020	1. Incomplete initial TURB 2. **TaHG tumors** 3. T1 tumors	1. Strong 2. Moderate 3. Strong	within 6 weeks
Chinese Urological Association	2019	1. Incomplete initial TURB 2. No muscle in specimen except for Ta LG/Gl and primary CIS 3. T1 tumors.	Moderate	2–6 weeks
SIU & International Consultation on Bladder Cancer (ICUD) 2017	2017	1. Incomplete initial resection 2. **TaHG tumors, particularly for patients with large or multifocal tumors** 3. T1 disease	1. B ** 2. C ** 3. B **	within 6 weeks
National Institute for Clinical Excellence (NICE)	2015	1. **All high-risk non-muscle invasive bladder cancer**	1. Low	within 6 weeks

The bold text represents the differences from EAU guidelines. TURB: transurethral resection of bladder tumor; CIS carcinoma in situ; LG: low grade; HG: high grade; * NCCN Categories of Evidence and Consensus; ** recommendation grades of Oxford Centre for Evidence-based Medicine.

**Table 2:** Characteristics and outcomes of included studies.

First Author & Year	Country	Study Type	Study Period	Re TURB Time	Participants	Groups	Patient Number	Male/ Female	Age Mean ± SD (Range) /Median (IQR)	Stage Ta/T1/Tis	Grade LG/HG	Residual Tumor (%)	Up Stage (%)	Recurrence (%)	RFS	Progression	PFS
Zhou 2020 [32]	China	RC	June 2012–June 2018	Within 2–6 weeks	Primary T1 and HG/G3 tumors, excluding primary CIS.	ReTURB	108	86/22	66.12 ± 1.52	60/48/0	25/83	6 (5.6)	2 (1.85)	23 (21.3)	1 year: 92.6 2 year: 88.4 * 3 year: 84.3 5 year: 68.0 *	4 (3.7)	1 year: 98.1 3 year: 96.3
						Control	143	111/32	68.59 ± 1.36	87/56/0	49/94	11 (7.69)	2 (1.40)	39 (27.2)	1 year: 90.2 2 year: 84.2 * 3 year: 80.4 5 year: 54.1 *	7 (4.9)	1 year: 97.9 3 year: 95.1
Xu 2021 [36]	China	RC	June 2015–June 2019	Within 6 weeks	Primary T1/TaHG tumors, Tumor number ≤ 4 Diameter ≤ 4 cm	ReTURB	51	41/10	67.4 ± 9.5	16/35/0	13/38	3 (5.88)	0 (0)	10 (19.6)	1 year: 92.2 2 year: 87.6 * 3 year: 81.1 * 5 year: 71.5 *	2 (3.9)	NA
						Control	64	53/11	66.8 ± 9.0	15/49/0	10/54	2 (3.13)	0 (0)	18 (28.1)	1 year: 90.6 2 year: 81.1 * 3 year: 66.4 * 5 year: 63.1 *	1 (1.5)	NA
Yanagisawa 2022 [38]	Japan	RC	April 2013–February 2021	Within 2–6 weeks	T1 Tumors	ReTURB	50	33/17	74 (70.25–78)	0/50/0	0/50	9 (18.0)	0 (0)	18 (36.0)	1 year: 66.5 * 2 year: 55.1 3 year: 54.9 * 5 year: 54.9 *	7 (14.0)	1 year: 95.7 * 3 year: 80.6 5 year: 64.5 *
						Control	56	43/13	76 (69–82.25)	0/56/0	0/56	NA	NA	18 (32.1)	1 year: 71.3 * 2 year: 59.9 3 year: 59.9 * 5 year: 54.0 *	6 (10.7)	1 year: 95.7 * 3 year: 82.6 5 year: 82.6 *
Wolters 2011 [27]	Germany	CS	June 2010–October 2010	Within 6 weeks	Solitary papillary lesions, treatment-naive, on the lower bladder wall and trigonum	ReTURB	5	4/1	57 (57–80)	2/3/0	G1 1 G2 1 G3 3	NA	NA	NA	NA	NA	NA
Muto 2014 [28]	Italy	PCS	April 2011–September 2012	Within 30–90 days	Naïve NMIBC	ReTURB	48	NA	NA	31/17/0	31/17/0	0 (0)	0 (0)	7 (14.6)	1.5 year: 85.4	0 (0)	NA
Migliari 2015 [29]	Italy	PC	February 2012–September 2013	Within 90 days	Single papillary bladder tumor, diameter ≥ 1 cm	ReTURB	53	NA	NA	30/23/0	30/23	0 (0)	0 (0)	12 (22.6)	1.5 year: Ta 90.0 T1 76.0	0 (0)	NA
Hurle 2020 [30]	Italy	RCS	September 2011–April 2017	Within 40 days	First diagnosis or a primary recurrence of High-risk NMIBC, a single tumor of ≤3 cm and ≤4 lesions	ReTURB	78	51/27	68 ± 9	17/57/4	G3 72	5 (6.41)	0 (0)	11 (14.1)	1 year: 93.4 * 2 year: 92.0 * 3 year: 85.0 * 5 year: 85.0 *	1 (1.3)	NA
Yang 2020 [31]	China	PC	October 2015–June 2017	Within 2–6 weeks	Primary, HG and/or T1 tumor; diameter between 1.0 to 3.0 cm	ReTURB	28	NA	NA	NA	NA	2 (7.14)	1 (3.57)	NA	NA	NA	NA
Hashem 2021 [33]	Egypt	RCT	September 2015–September 2018	4 weeks after the primary resection	NMIBC	ReTURB	44	NA	NA	2/42	28/16	3 (6.82)	0 (0)	7 (15.9)	1 year: 92.6 * 2 year: 80.0 * 3 year: 80.0 *	NA	NA
Hu 2021 [34]	China	RCS	January 2019–October 2019	4–6 weeks	Primary T1 or TaHG	ReTURB	10	NA	NA	NA	NA	0 (0)	0 (0)	NA	NA	NA	NA
Poletajew 2021 [35]	Poland	PC	NA	Within 2–6 weeks	1–4 cm in diameter.	ReTURB	37	NA	NA	NA	NA	11 (29.73)	NA	NA	NA	NA	NA
Fan 2022 [37]	China	RCS	2013– 2019	Within 6 weeks	NA	ReTURB	27	NA	NA	NA	NA	4 (14.81)	NA	NA	NA	NA	NA

CS: case series; HG: high grade; IQR: interquartile range; LG: low grade; NA: not available; NMIBC: non-muscle invasive bladder cancer; PC: prospective cohort; PCS: prospective case series; RC: retrospective cohort; RCS: retrospective case series; reTURB: repeat transurethral resection of bladder tumor; RFS: recurrence-free survival; PFS: progression-free survival; SD: standard deviation; * Digitized from the Kaplan—Meier plots.

## Data Availability

Not applicable.

## Supplementary Materials

The following supporting information can be downloaded at: https://www.mdpi.com/article/10.3390/jcm11175049/s1, Table S1. NOS scores of included studies. Table S2. Risk of bias assessment of included studies. Table S3. Egger test and Begg’s test for pooled comparisons and sensitivity analysis by trim and fill method. Figure S1. Funnel plots of sensitivity analysis.

## References

[1] Richters A., Aben K.K.H., Kiemeney L. (2020). The global burden of urinary bladder cancer: An update. World J. Urol..

[2] Safiri S., Kolahi A.A., Naghavi M., Global Burden of Disease Bladder Cancer Collaborators (2021). Global, regional and national burden of bladder cancer and its attributable risk factors in 204 countries and territories, 1990–2019: A systematic analysis for the Global Burden of Disease study 2019. BMJ Glob. Health.

[3] Babjuk M., Burger M., Capoun O., Cohen D., Comperat E.M., Dominguez Escrig J.L., Gontero P., Liedberg F., Masson-Lecomte A., Mostafid A.H. (2022). European Association of Urology Guidelines on Non-muscle-invasive Bladder Cancer (Ta, T1, and Carcinoma in Situ). Eur. Urol..

[4] Kramer M.W., Altieri V., Hurle R., Lusuardi L., Merseburger A.S., Rassweiler J., Struck J.P., Herrmann T.R.W. (2017). Current Evidence of Transurethral En-bloc Resection of Nonmuscle Invasive Bladder Cancer. Eur. Urol. Focus.

[5] Ukai R., Kawashita E., Ikeda H. (2000). A new technique for transurethral resection of superficial bladder tumor in 1 piece. J. Urol..

[6] Yanagisawa T., Mori K., Motlagh R.S., Kawada T., Mostafaei H., Quhal F., Laukhtina E., Rajwa P., Aydh A., Konig F. (2022). En Bloc Resection for Bladder Tumors: An Updated Systematic Review and Meta-Analysis of Its Differential Effect on Safety, Recurrence and Histopathology. J. Urol..

[7] Symeonidis E.N., Lo K.L., Chui K.L., Vakalopoulos I., Sountoulides P. (2022). En bloc resection of bladder tumors: Challenges and unmet needs in 2022. Future Oncol..

[8] Teoh J.Y., MacLennan S., Chan V.W., Miki J., Lee H.Y., Chiong E., Lee L.S., Wei Y., Yuan Y., Yu C.P. (2020). An International Collaborative Consensus Statement on En Bloc Resection of Bladder Tumour Incorporating Two Systematic Reviews, a Two-round Delphi Survey, and a Consensus Meeting. Eur. Urol..

[9] Powles T., Bellmunt J., Comperat E., De Santis M., Huddart R., Loriot Y., Necchi A., Valderrama B.P., Ravaud A., Shariat S.F. (2022). Bladder cancer: ESMO Clinical Practice Guideline for diagnosis, treatment and follow-up. Ann. Oncol..

[10] Chang S.S., Boorjian S.A., Chou R., Clark P.E., Daneshmand S., Konety B.R., Pruthi R., Quale D.Z., Ritch C.R., Seigne J.D. (2016). Diagnosis and Treatment of Non-Muscle Invasive Bladder Cancer: AUA/SUO Guideline. J. Urol..

[11] Monteiro L.L., Witjes J.A., Agarwal P.K., Anderson C.B., Bivalacqua T.J., Bochner B.H., Boormans J.L., Chang S.S., Dominguez-Escrig J.L., McKiernan J.M. (2019). ICUD-SIU International Consultation on Bladder Cancer 2017: Management of non-muscle invasive bladder cancer. World J. Urol..

[12] Bhindi B., Kool R., Kulkarni G.S., Siemens D.R., Aprikian A.G., Breau R.H., Brimo F., Fairey A., French C., Hanna N. (2021). Canadian Urological Association guideline on the management of non-muscle-invasive bladder cancer—Abridged version. Can. Urol. Assoc. J..

[13] NCC Networks Inc. NCCN Clinical Practice Guidelines in Oncology: Bladder Cancer (Version 2 2022). https://www.nccn.org/professionals/physician_gls/pdf/bladder.pdf.

[14] Huang J., Xu C., Zhang X., Huang J. (2020). Chinese Urological Association guidelines on the diagnosis and treatment of bladder cancer. Chinese Guidelines on Urological and Andrological Disease.

[15] NICE Guideline: Bladder Cancer: Diagnosis and Management. https://www.nice.org.uk/guidance/ng2.

[16] Kim L.H.C., Patel M.I. (2020). Transurethral resection of bladder tumour (TURBT). Transl. Androl. Urol..

[17] Divrik R.T., Sahin A.F., Yildirim U., Altok M., Zorlu F. (2010). Impact of routine second transurethral resection on the long-term outcome of patients with newly diagnosed pT1 urothelial carcinoma with respect to recurrence, progression rate, and disease-specific survival: A prospective randomised clinical trial. Eur. Urol..

[18] Sfakianos J.P., Kim P.H., Hakimi A.A., Herr H.W. (2014). The effect of restaging transurethral resection on recurrence and progression rates in patients with nonmuscle invasive bladder cancer treated with intravesical bacillus Calmette-Guerin. J. Urol..

[19] Rubio-Briones J., Algaba F., Gallardo E., Marcos-Rodriguez J.A., Climent M.A., on Behalf of the Sogug Multidisciplinary Working Group (2021). Recent Advances in the Management of Patients with Non-Muscle-Invasive Bladder Cancer Using a Multidisciplinary Approach: Practical Recommendations from the Spanish Oncology Genitourinary (SOGUG) Working Group. Cancers.

[20] Del Giudice F., Flammia R.S., Chung B.I., Moschini M., Pradere B., Mari A., Soria F., Albisinni S., Krajewski W., Szydelko T. (2022). Compared Efficacy of Adjuvant Intravesical BCG-TICE vs. BCG-RIVM for High-Risk Non-Muscle Invasive Bladder Cancer (NMIBC): A Propensity Score Matched Analysis. Cancers.

[21] Del Giudice F., Busetto G.M., Gross M.S., Maggi M., Sciarra A., Salciccia S., Ferro M., Sperduti I., Flammia S., Canale V. (2021). Efficacy of three BCG strains (Connaught, TICE and RIVM) with or without secondary resection (re-TUR) for intermediate/high-risk non-muscle-invasive bladder cancers: Results from a retrospective single-institution cohort analysis. J. Cancer Res. Clin. Oncol..

[22] Lee L.J., Kwon C.S., Forsythe A., Mamolo C.M., Masters E.T., Jacobs I.A. (2020). Humanistic and Economic Burden of Non-Muscle Invasive Bladder Cancer: Results of Two Systematic Literature Reviews. Clin. Outcomes Res..

[23] Page M.J., McKenzie J.E., Bossuyt P.M., Boutron I., Hoffmann T.C., Mulrow C.D., Shamseer L., Tetzlaff J.M., Akl E.A., Brennan S.E. (2021). The PRISMA 2020 statement: An updated guideline for reporting systematic reviews. BMJ.

[24] Wells G., Shea B., O’Connell D., Peterson J., Welch V., Losos M., Tugwell P. The Newcastle-Ottawa Scale (NOS) for Assessing the Quality of Nonrandomised Studies in Meta-Analyses. http://www.ohri.ca/programs/clinical_epidemiology/oxford.asp.

[25] Knoll T., Omar M.I., Maclennan S., Hernandez V., Canfield S., Yuan Y., Bruins M., Marconi L., Van Poppel H., N’Dow J. (2018). Key Steps in Conducting Systematic Reviews for Underpinning Clinical Practice Guidelines: Methodology of the European Association of Urology. Eur. Urol..

[26] Tierney J.F., Stewart L.A., Ghersi D., Burdett S., Sydes M.R. (2007). Practical methods for incorporating summary time-to-event data into meta-analysis. Trials.

[27] Wolters M., Kramer M.W., Becker J.U., Christgen M., Nagele U., Imkamp F., Burchardt M., Merseburger A.S., Kuczyk M.A., Bach T. (2011). Tm:YAG laser en bloc mucosectomy for accurate staging of primary bladder cancer: Early experience. World J. Urol..

[28] Muto G., Collura D., Giacobbe A., D’Urso L., Muto G.L., Demarchi A., Coverlizza S., Castelli E. (2014). Thulium:yttrium-aluminum-garnet laser for en bloc resection of bladder cancer: Clinical and histopathologic advantages. Urology.

[29] Migliari R., Buffardi A., Ghabin H. (2015). Thulium laser endoscopic en bloc enucleation of nonmuscle-invasive bladder cancer. J. Endourol..

[30] Hurle R., Casale P., Lazzeri M., Paciotti M., Saita A., Colombo P., Morenghi E., Oswald D., Colleselli D., Mitterberger M. (2020). En bloc re-resection of high-risk NMIBC after en bloc resection: Results of a multicenter observational study. World J. Urol..

[31] Yang Y., Liu C., Yang X., Wang D. (2020). Transurethral en bloc resection with monopolar current for non-muscle invasive bladder cancer based on TNM system. Transl. Cancer Res..

[32] Zhou W., Wang W., Wu W., Yan T., Du G., Liu H. (2020). Can a second resection be avoided after initial thulium laser endoscopic en bloc resection for non-muscle invasive bladder cancer? A retrospective single-center study of 251 patients. BMC Urol..

[33] Hashem A., Mosbah A., El-Tabey N.A., Laymon M., Ibrahiem E.-H., Abd Elhamid M., Elshal A.M. (2021). Holmium Laser En-bloc Resection Versus Conventional Transurethral Resection of Bladder Tumors for Treatment of Non-muscle-invasive Bladder Cancer: A Randomized Clinical Trial. Eur. Urol. Focus.

[34] Hu H., Li B., Liu Z., Meng X., Li C., Li F., Hu J., Chen Y., Li Z., Wang S. (2021). The individual surgical protocol of transurethral en bloc resection of bladder tumor based on VI-RADS and preliminary experience. Chin. J. Urol..

[35] Poletajew S., Krajewski W., Stelmach P., Adamowicz J., Nowak L., Moschini M., Zapala P., Drewa T., Paradysz A., Radziszewski P. (2021). En-bloc resection of urinary bladder tumour—A prospective controlled multicentre observational study. Videosurg. Other Miniinvasive Tech..

[36] Xu S., Cao P., Wang K., Wu T., Hu X., Chen H., Xu L., Gu J., Wu S., Zhu L. (2021). Clinical Outcomes of Reresection in Patients with High-Risk Nonmuscle-Invasive Bladder Cancer Treated with en Bloc Transurethral Resection: A Retrospective Study with a 1-Year Follow-Up. J. Endourol..

[37] Fan J., Zhang X., Fan J., Li L., He D., Wu K. (2022). Risk Stratification for the Rate and Location of Residual Bladder Tumor for the Decision of Re-Transurethral Resection of Bladder Tumor. Front. Oncol..

[38] Yanagisawa T., Sato S., Hayashida Y., Okada Y., Iwatani K., Matsukawa A., Kimura T., Takahashi H., Egawa S., Shariat S.F. (2022). Do we need repeat transurethral resection after en bloc resection for pathological T1 bladder cancer?. BJU Int..

[39] Cumberbatch M.G.K., Foerster B., Catto J.W.F., Kamat A.M., Kassouf W., Jubber I., Shariat S.F., Sylvester R.J., Gontero P. (2018). Repeat Transurethral Resection in Non-muscle-invasive Bladder Cancer: A Systematic Review. Eur. Urol..

[40] Soria F., Marra G., D’Andrea D., Gontero P., Shariat S.F. (2019). The rational and benefits of the second look transurethral resection of the bladder for T1 high grade bladder cancer. Transl. Androl. Urol..

[41] Soria F., Giordano A., Gontero P. (2020). Transurethral resection of bladder tumor and the need for re-transurethral resection of bladder tumor: Time to change our practice?. Curr. Opin. Urol..

[42] Creta M., Celentano G., Califano G., La Rocca R., Longo N. (2022). En-bloc Laser Resection of Bladder Tumors: Where Are We Now?. J. Clin. Med..

[43] Croghan S.M., Compton N., Manecksha R.P., Cullen I.M., Daly P.J. (2022). En bloc transurethral resection of bladder tumors: A review of current techniques. Can. Urol. Assoc. J..

[44] Lin L., Guo X., Ma Y., Zhu J., Li X. (2022). Does repeat transurethral resection of bladder tumor influence the diagnosis and prognosis of T1 bladder cancer? A systematic review and meta-analysis. Eur. J. Surg. Oncol..

[45] Wong V.K., Ganeshan D., Jensen C.T., Devine C.E. (2021). Imaging and Management of Bladder Cancer. Cancers.

