# Supplementary material for: A Systematic Review on the Role of Repeat Transurethral Resection after Initial en Bloc Resection for Non-Muscle Invasive Bladder Cancer
Source: J Clin Med. 2022 Aug 28;11(17):5049. doi: 10.3390/jcm11175049 (PMC9456573; doi:10.3390/jcm11175049)
Supplement: Supplementary file 1 [file jcm-11-05049-s001.zip › jcm-1881733-supplementary.pdf]

Journal of
Clinical Medicine

MDPI

Table S1. NOS scores of included studies.

Study	Zhou	Xu	Yanagisawa
Representiveness of the exposed cohort	1	1	1
Selection of the non exposed cohort	1	1	1
Ascertainment of exposure	1	1	1
Demonstration that outcome of interest was not present at start of study	1	1	1
Comparability of cohorts on the basis of the design or analysis	2	2	2
Assessment of outcome	1	1	1
Is follow up long enough for outcomes to occur	1	1	1
Adequacy of follow up of cohorts	1	0	0
NOS score	9	8	8

Table S2. Risk of bias assessment of included studies.

Study	1. Was there an a priori protocol?	2. Was the total population included or were study participants selected consecutively?	3. Was outcome data complete for all participants and any missing data adequately explained/unlikely to be related to the outcome?	4. Were all prespecified outcomes of interest and expected outcomes reported?	5. Were primary benefit and harm outcomes appropriately measured?	Risk of Bias assessment
Wolters	unspecified	unspecified	Yes	Yes	No	High
Muto	Yes	unspecified	Yes	Yes	Yes	High
Migliari	Yes	unspecified	Yes	Yes	Yes	High
Hurle	Yes	unspecified	Yes	Yes	Yes	High
Yang	Yes	unspecified	Yes	Yes	Yes	High
Hashem	Yes	Yes	Yes	Yes	Yes	Low
Hu	Yes	No	Yes	Yes	Yes	High
Poletajew	Yes	Yes	Yes	Yes	No	High
Fan	unspecified	unspecified	Yes	Yes	No	High

Table S3. Egger test and Begg's test for pooled comparisons and sensitivity analysis by trim and fill method.

Comparisons	Egger test		Begg's test		Hazard Ratio /Relative Risk	imputed study number	Adjusted Hazard Ratio/Relative Risk by Trim and fill method
	Z	p	Z	p			
1-year RFS	-0.34	0.7306	-2.09	0.2963	0.74(0.36-1.51)	2	0.86(0.48-1.53)
2-year RFS	-0.38	0.7045	-2.09	0.2963	0.76(0.45-1.26)	2	0.82(0.54-1.24)
3-year RFS	-0.03	0.9753	-2.09	0.2963	0.83(0.53-1.32)	2	0.84(0.58-1.21)
5-year RFS	0.31	0.7544	1.04	0.2963	0.79(0.66-1.23)	2	0.79(0.58-1.09)
Recurrence	-0.08	0.935	-1.04	1	0.88(0.64-1.21)	0	0.88(0.64-1.21)
Progression	0.54	0.5916	0	1	1.12(0.53-2.35)	1	1.03(0.50-2.08)

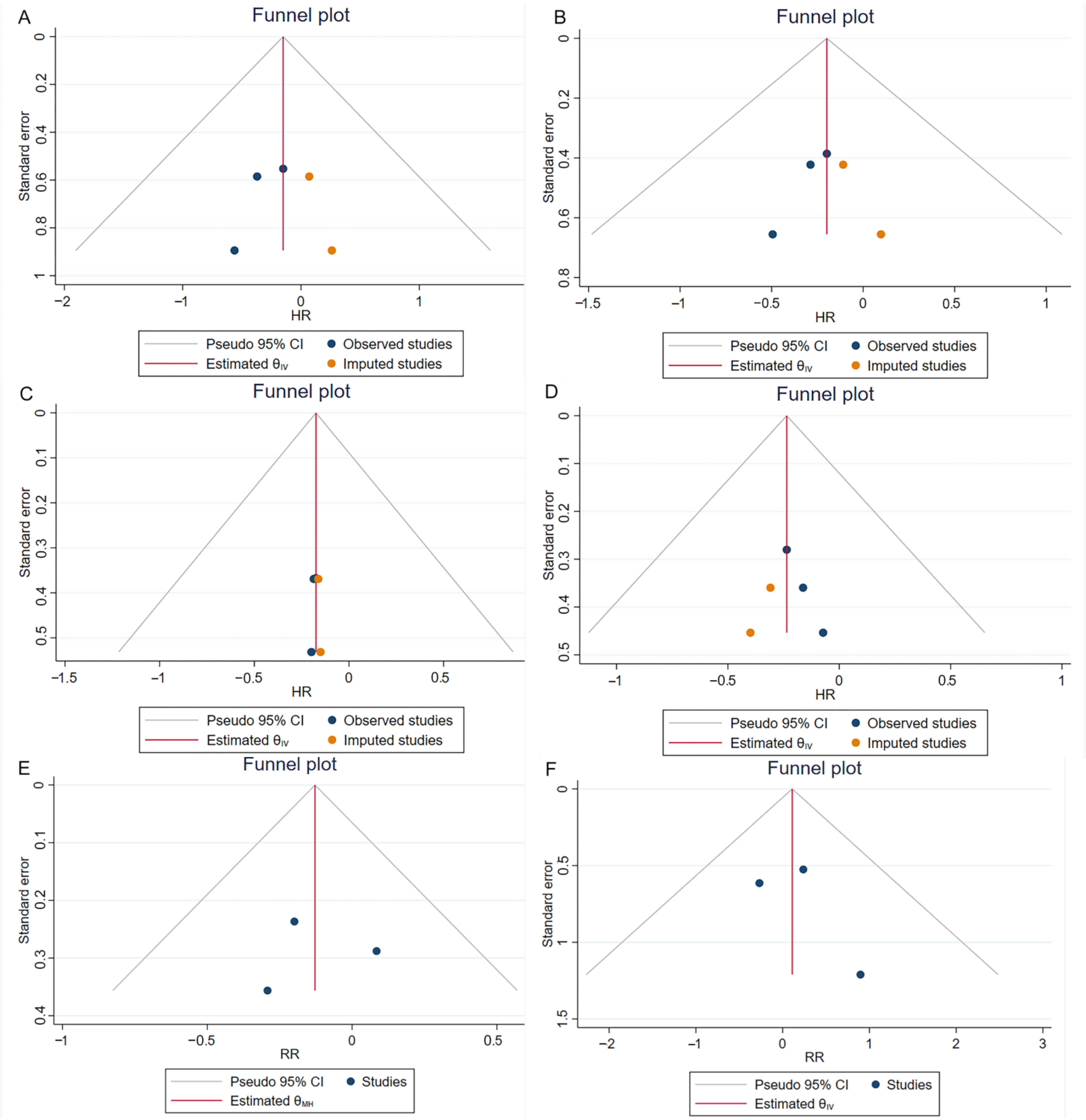

Figure S1. Funnel plots of sensitivity analysis for comparisons 1-year RFS (A), 2-year RFS (B), 3-year RFS (C), 5-year RFS (D), recurrence (E) and progression (F) between reTURB group and control group.
